# Zinc Oxide Nanorods-Decorated Graphene Nanoplatelets: A Promising Antimicrobial Agent against the Cariogenic Bacterium *Streptococcus mutans*

**DOI:** 10.3390/nano6100179

**Published:** 2016-09-29

**Authors:** Elena Zanni, Chandrakanth Reddy Chandraiahgari, Giovanni De Bellis, Maria Rita Montereali, Giovanna Armiento, Paolo Ballirano, Antonella Polimeni, Maria Sabrina Sarto, Daniela Uccelletti

**Affiliations:** 1 BBCD, Department of Biology and Biotechnology, Sapienza University of Rome, Rome 00185, Italy; elena.zanni@uniroma1.it; 2 SNN Lab, Sapienza Nanotechnology & Nano-Science Laboratory, Sapienza University of Rome, Rome 00185, Italy; c.chandraiahgari@uniroma1.it (C.R.C.); giovanni.debellis@uniroma1.it (G.D.B.); 3 DIAEE, Department of Astronautical, Electrical, Energy Engineering, Sapienza University of Rome, Rome 00185, Italy; 4 Sustainable Territorial and Production Systems Department (SSPT) PROTER Division, BioGeoChemistry Laboratory, ENEA, National Agency for New Technologies, Energy and Sustainable Economic Development, Rome 00123, Italy; mariarita.montereali@enea.it (M.R.M.); giovanna.armiento@enea.it (G.A.); 5 Department of Earth Science, Sapienza University of Rome, Rome 00185, Italy; paolo.ballirano@uniroma1.it; 6 Department of Dentistry and Maxillo-Facial Sciences, Unit of Pediatric Dentistry Sapienza University of Rome, Rome 00185, Italy; antonella.polimeni@uniroma1.it

**Keywords:** *streptococcus mutans*, antimicrobial activity, graphene nanoplatelets, zinc oxide, composite, dental caries

## Abstract

Nanomaterials are revolutionizing the field of medicine to improve the quality of life due to the myriad of applications stemming from their unique properties, including the antimicrobial activity against pathogens. In this study, the antimicrobial and antibiofilm properties of a novel nanomaterial composed by zinc oxide nanorods-decorated graphene nanoplatelets (ZNGs) are investigated. ZNGs were produced by hydrothermal method and characterized through field-emission scanning electron microscopy (FE-SEM), energy-dispersive X-ray spectroscopy (EDX) and X-ray diffraction (XRD) techniques. The antimicrobial activity of ZNGs was evaluated against *Streptococcus mutans*, the main bacteriological agent in the etiology of dental caries. Cell viability assay demonstrated that ZNGs exerted a strikingly high killing effect on *S. mutans* cells in a dose-dependent manner. Moreover, FE-SEM analysis revealed relevant mechanical damages exerted by ZNGs at the cell surface of this dental pathogen rather than reactive oxygen species (ROS) generation. In addition, inductively coupled plasma mass spectrometry (ICP-MS) measurements showed negligible zinc dissolution, demonstrating that zinc ion release in the suspension is not associated with the high cell mortality rate. Finally, our data indicated that also *S. mutans* biofilm formation was affected by the presence of graphene-zinc oxide (ZnO) based material, as witnessed by the safranin staining and growth curve analysis. Therefore, ZNGs can be a remarkable nanobactericide against one of the main dental pathogens. The potential applications in dental care and therapy are very promising.

## 1. Introduction

Dental caries represent an increasingly serious health problem for which *Streptococcus mutans* has been identified as the main etiologic cause (reviewed in [1]). Nowadays, attention is focused on the development of suitable materials able to kill or inhibit this bacterium and, thus, to control the pathologic condition.

Although antimicrobial compounds have been reported to decrease the occurrence of dental diseases, the use of antibiotics as well as chemical bactericides can impact negatively on the bacterial flora of oral cavity and intestine tract [2,3]. Since pathogens are able to acquire resistance against different antibiotics, agents characterized by a notable antibacterial activity and that do not develop resistance are now highly requested [4]. Based on that, nanotechnology is considered a powerful tool. During the last few years, ever-growing attention was focused on metals nanoparticles (i.e., silver and zinc) due to their remarkable antimicrobial properties [5]. The high antibacterial effect of these nanostructured agents is ascribed to the high surface area to volume ratio, enabling greater presence of atoms on the surface, and thus providing maximum contact with the environment. Because of their capability to easily penetrate cell membranes, several intracellular processes are disrupted resulting in high reactivity and antibacterial activity [6].

Graphitic nanomaterials, including carbon nanotubes (CNTs), fullerenes, and graphene, are considered as novel and very promising agents due to their innovative features, including antibacterial properties [7,8,9]. Graphene, a two-dimensional mono-atomic thick material with sp^2^ hybridized carbon arrangement, has attracted extensive attention during the past decade. Its unique and outstanding electrical conductivity, mechanical properties, large surface area, low coefficient of thermal expansion, and very high aspect ratio make it very attractive for several potential applications in many different fields [10,11,12,13,14]. Moreover, graphene is biocompatible and it is a suitable substrate for biological/chemical functionalizations [15,16]. Similar to CNTs, graphene-based materials have received significant attention for their potential applications in the biological/medical field, including bacterial inhibition, drug delivery, and photothermal cancer therapy [17,18,19]. In this context, graphene-related structures like graphene nanoplatelets (GNPs), can represent a valuable tool in the biological/medical field, also owing to the fact that their production process is very easy, inexpensive, and scalable [12]. The antimicrobial properties of GNPs against both gram-negative (*Pseudomonas aeruginosa*) and gram-positive (*S. mutans*) bacteria have been investigated in previous studies [20,21], and their very low cytotoxicity was also demonstrated through in vivo system (*Caenorhabditis elegans*) [20]. However, one of the main limitations for a wide exploitation of GNPs as antimicrobial agent in dental application, is represented by the grey color and by the aptitude in aggregating when dispersed in a colloidal suspension.

Metal oxides are largely utilized in the field of nanotechnology; among them, zinc oxide (ZnO), a wide band-gap II–VI semiconductor, has attracted growing interest due to its unique optical, luminescent, electronic, optoelectronic, and biocompatible properties [22,23]. Several methods have been developed to synthesize ZnO materials as one-dimensional (1D) nanostructures with different morphologies including nanowires, nanorods, nanoneedles, and nanorings [24,25,26,27,28]. Synthesis of ZnO nanorods (ZnO-NRs) via chemical approaches opens the route to low-cost catalyst-free mass-production of ZnO nanostructures [29,30,31,32]. In our previous studies, through both in vitro and in vivo systems, we have demonstrated the very low cytotoxicity of ZnO-NRs [33], together with their great potential as antibacterial material acting as nano-needles against *Staphylococcus aureus* and *Bacillus subtilis* [34].

In the present work, we aim to propose the original use of a novel hybrid material, featured by ZnO-NRs grown on multilayer graphene sheets (i.e., GNPs), as antimicrobial nanomaterial against *S. mutans*, combining the antimicrobial effect of GNPs with the light color and biocidal properties of ZnO-NRs.

ZnO-NRs-decorated GNPs (ZNGs) are a novel class of engineered nanomaterials in which pristine GNPs are densely decorated with ZnO-NRs through a facile hydrothermal method [35]. In this study, the intriguing antimicrobial activity of ZNGs dispersed in water was compared with the one of colloidal suspensions containing either pristine GNPs or ZnO-NRs or both GNPs and ZnO-NRs. The final goal is to reveal the potential applications of ZNGs, having strikingly high antimicrobial properties, in the dental material field.

## 2. Results and Discussion

### 2.1. Morphological and Structural Properties

**Figure 1:**
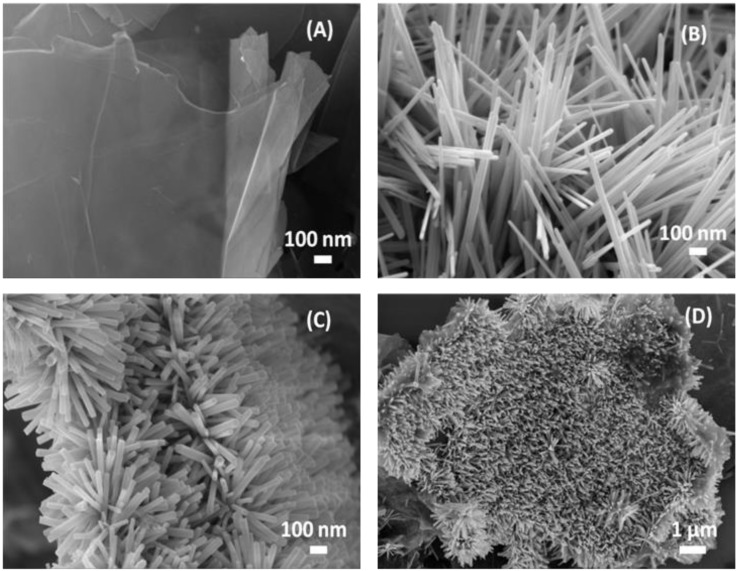
Field emission scanning electron microscopy (FE-SEM) images of (**A**) pristine graphene nanoplatelets (GNPs), (**B**) pristine zinc oxide nanorods (ZnO-NRs), and (**C**,**D**) ZnO-NRs-decorated GNPs (ZNGs).

Pristine GNPs, pristine ZnO-NRs and hybrid ZNG nanostructures were produced in water-based colloidal suspension at Sapienza Nanotechnology and Nanoscience Laboratory (SNN-Lab) (as described in Section 3.2). The morphology of the produced nanomaterials was investigated through high-resolution field emission scanning electron microscopy (FE-SEM) (Figure 1). GNPs (Figure 1A) are made of multiple graphene sheets staked in 2D-platelets having thickness in the range of 2–10 nm and average lateral dimensions in the range of 1–10 µm [20]. ZnO-NRs (Figure 1B) are straight rods of ZnO having a hexagonal cross section, with average diameter of ~36 nm and length in the range of 400 nm–1 µm. Figure 1C,D show the surface morphology of ZNGs: GNPs are densely decorated with ZnO-NRs having average diameter of ~34 nm and length of 300–400 nm.

**Figure 2:**
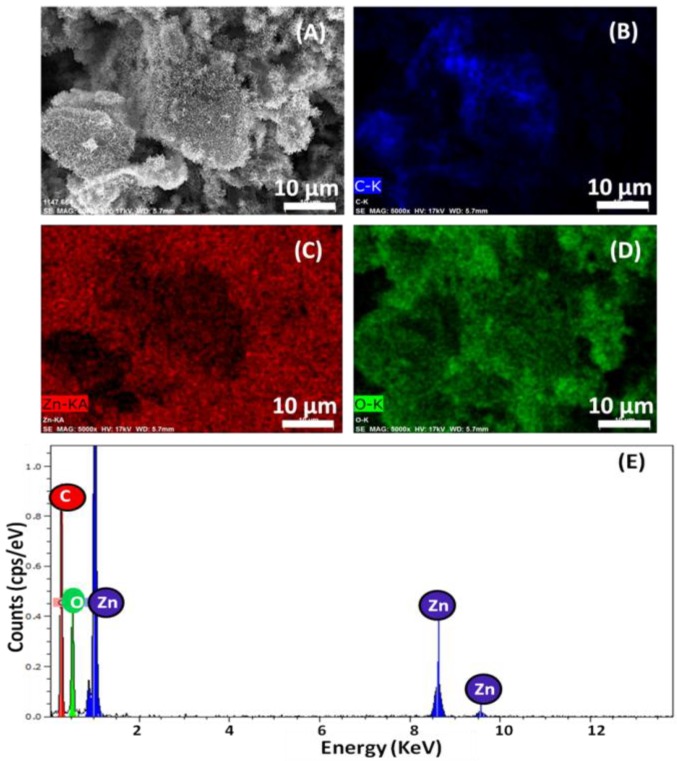
Energy-dispersive X-ray spectroscopy (EDX) elemental analysis performed on the (**A**) ZNGs and elemental mapping for (**B**) carbon, (**C**) zinc, (**D**) oxygen, and (**E**) corresponding EDX spectrum.

Figure 2A–E show the elemental analysis and typical energy-dispersive X-ray spectroscopy (EDX) spectrum obtained for ZNGs. The elemental analysis of ZNGs was performed in the specific area shown in Figure 2A and revealed that only carbon (C), zinc (Zn), and oxygen (O) signals were detected (Figure 2B–D). No other signal of secondary phase or impurity was detected as shown in Figure 2E. This indicates the high purity chemical composition of the ZNGs used in this study. The elemental mapping also demonstrated that the GNPs are densely decorated with ZnO-NRs.

**Figure 3:**
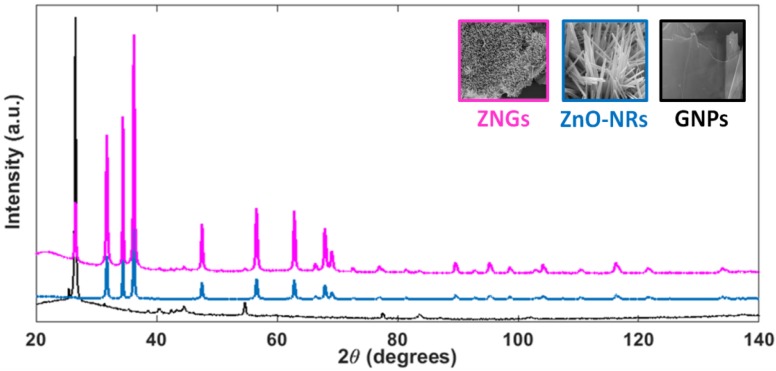
X-ray diffraction patterns of all the produced nanostructures.

The X-ray diffraction (XRD) patterns of GNPs, ZnO-NRs and ZNGs are shown in Figure 3. The peak positions of the obtained spectra for samples of ZnO-NRs and ZNGs are in excellent agreement with the published Joint Committee on Powder Diffraction Standards (JCPDS) card 036-1451 of wurtzite structure of ZnO, with lattice constants a = 3.25 Å and c = 5.17 Å. The spectrum of the pristine GNPs consists of a strong graphitic peak at its 2θ value of 26.53° and in secondary peaks from 40° to 60° [36]. The same peaks are present in the XRD pattern of sample ZNGs. No diffraction peaks of impurity were detected, suggesting that the synthesized nanomaterials are of high-purity. Further, the sharp and intense diffraction peaks indicate the highly crystalline nature of the produced nanostructures.

### 2.2. Antimicrobial Activity

In the last years, our attention has been focused towards the study of the antibacterial properties characterizing graphene- and metal oxide-based nanomaterials, such as GNPs and ZnO-NRs, respectively [20,34]. In order to compare the killing activity of GNPs and ZnO-NRs versus oral pathogen bacteria, the antimicrobial potential of such nanomaterials was firstly evaluated on *S. mutans* cells. Among the 500 different bacterial species identified in the oral cavity, *S. mutans* is the most commonly isolated [37], and like other oral streptococci it is considered as the primary plaque-former and is involved in plaque formation and initiation of dental caries [38,39].

**Figure 4:**
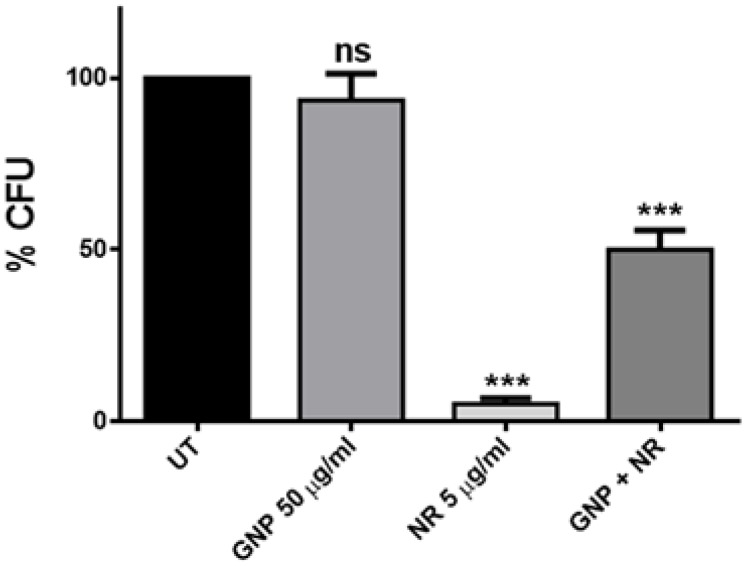
Cell survival after treatment with GNPs, ZnO-NRs, and the combination of both materials. Statistical analysis was performed by one-way analysis of variance (ANOVA) method coupled with the Bonferroni post-test (ns not significant; *** *p* < 0.001 compared to the control).

As shown in Figure 4, ZnO-NRs displayed a strikingly bactericidal effect (up to 95% of cell viability reduction) on *S. mutans* cells, even at a very low concentration (5 µg/mL). This observation is in line with the strong antimicrobial potential reported for ZnO-NRs against *S. aureus* and *B. subtilis* that, like *S. mutans*, are members of the Gram-positive bacteria class [34]. Actually, due to their 1D-structure, ZnO-NRs act as nano-needles inducing serious damage of the cell membrane. 

By contrast, no effect on *S. mutans* viability was highlighted with GNPs treatment at the concentration of 50 µg/mL (Figure 4). Actually, graphene and graphene-related materials, such as GNPs, have been demonstrated to have a remarkable antibacterial effect against many microorganisms. In fact, in the last few years, Liu and coworkers reported the comparison of the antibacterial activities of four graphene-based materials towards *Escherichia coli* and found that graphene oxide (GO) dispersion showed the highest antibacterial activity [40]. In addition, we demonstrated that GNPs, produced from thermal exfoliation of graphite intercalation compound, have bactericidal effects against *P. aeruginosa* and *S. mutans* cells. This is mainly due to a mechanical interaction originated by the GNP wrapping around the cells and a local damage of the cell wall produced by the GNP sharp edges acting as nano-knives [20,21]. However, the main problem in the use of GNPs in water-based colloidal suspension is the formation of aggregates, which inhibit the antimicrobial action of the nanostructures. Therefore, the concentration of GNPs used in this study (50 µg/mL) was too low for killing *S. mutans* cells, probably due to the formation of large aggregates. Indeed, we previously reported a remarkable mortality rate for the planktonic form of this bacterium only at very high concentrations of GNPs [21].

In order to exploit the combined effect of GNPs and ZnO-NRs, we performed the vitality test using a two-phase colloidal suspension containing both nanomaterials (50 µg/mL of GNPs and 5 µg/mL of ZnO-NRs). This resulted in a 50% reduction of bacterial survival (Figure 4), revealing that the presence of the GNPs in the suspension may inhibit the killing action of ZnO-NRs as nano-needles.

**Figure 5:**
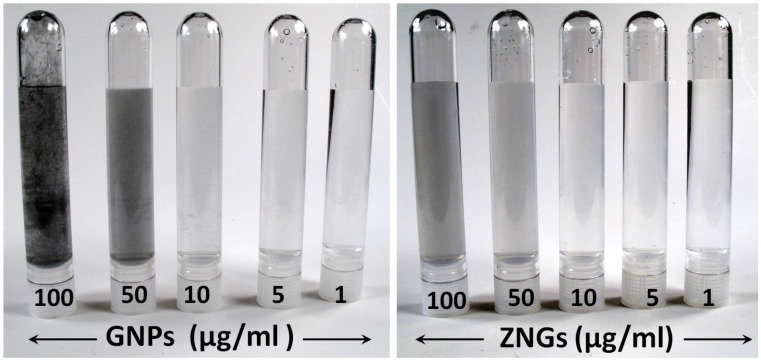
Photographs of GNPs and ZNGs aqueous suspensions prepared at various concentrations.

Starting from this, we produced ZNGs in which GNP offers a wide 2D-substrate for the oriented growth of ZnO-NRs nearly orthogonal to the platelet surface. Consequently, the new nanomaterial enables exploitation of the large-interaction area with bacterial cells offered by GNPs with the nano-needle action of ZnO-NRs. Moreover, ZnO-NRs-decoration of GNPs prevents GNP agglomerate formation in water-based suspension, and lightens the characteristic grey color of the carbon nanostructures at different concentrations (Figure 5), making ZNGs very promising for dental applications.

**Figure 6:**
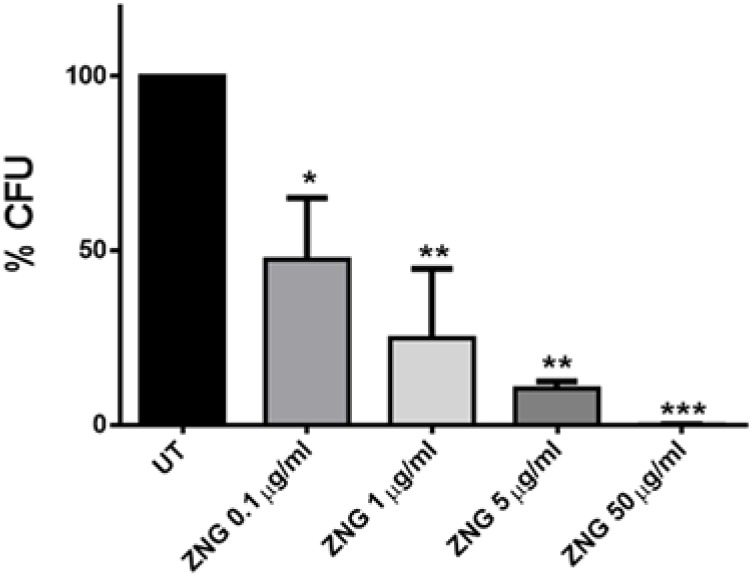
Concentration-dependent antibacterial activity of ZnO-NRs-decorated GNPs (ZNG) against bacteria cells. Loss of cell viability rate was obtained by colony counting method. Error bars represent the standard deviation. Statistical analysis was performed by one-way ANOVA method coupled with the Bonferroni post-test (ns not significant; * *p* < 0.05; ** *p* < 0.01; *** *p* < 0.001 compared to the control).

In order to evaluate whether this novel nanomaterial could be exploited to debate *S. mutans*, bacterial cells were challenged for 24 h with ZNGs. Remarkably, the exposure of the tested cells to ZNGs induced a relevant mortality rate in a dose-dependent manner (Figure 6). A 10% survival was pointed out when exposing cells to just 5 µg/mL, while a 99.9% bactericidal effect was observed at the highest concentration (50 µg/mL). *S. mutans* is of considerable clinical importance in dentistry, but compared to other species of microbes, there are relatively few reports on the effects of nanomaterials on this organism. The antimicrobial properties of metal-based nanoparticles have been highlighted by Espinosa-Cristobal et al. [41]. Moreover, it has been found that silver nanoparticles were more antibacterial to *S. mutans* than the traditional chlorhexidine disinfectant used in dentistry [42]. Furthermore, ZnO and copper oxide nanoparticles inhibited biofilm formation of *S. mutans* [43].

### 2.3. Field Emission Scanning Electron Microscopy Analysis of Cells Interaction with Zinc Oxide Nanorods-Decorated Graphene Nanoplatelets

**Figure 7:**
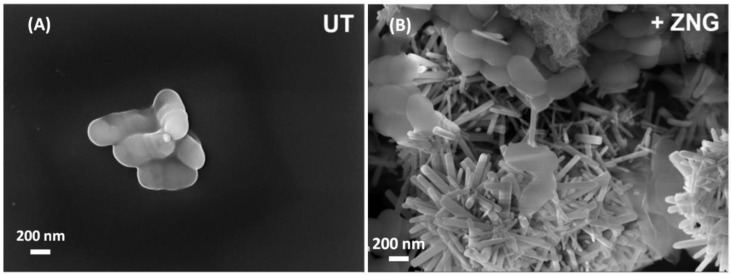
FE-SEM micrographs of *S.mutans* cells; (**A**) untreated and (**B**) treated with ZNGs suspension (50 µg/mL) for 24 h.

Since cellular mechanical damages can be caused by a direct contact between bacterial surface and graphene-based materials, a FE-SEM analysis was performed to examine the interactions between ZNGs and *S. mutans* cells. In Figure 7 it is shown how ZNGs contact and damage *S. mutans* cells by puncturing the cellular surface through ZnO-NRs that protrude from GNP sheets. Acting as a network of nanoneedles, ZNGs entrap and literally spear bacterial cells, therefore inducing severe mechanical damage.

We observed a remarkable bactericidal activity that may be ascribed to the synergic antimicrobial functionalities arising from an increased interaction between cell wall and nanostructures. The main killing mechanism may be attributed to the mechanical damage produced by the ZnO-NRs affecting the cell wall. More deeply, the large lateral size of the decorated GNPs together with the preferred growth orientation of the ZnO-NRs over the GNP surface contributes to increasing the adhesion of the nanostructures to the cell wall, and enhancing the penetration of the ZnO-NRs through the cell membrane. This, in turn, may lead to a higher capability of puncturing and damaging the bacterial surface. Notably, this effect is limited to bacterial cells; indeed our previous study showed no membrane damages in two different human cell lines treated with ZnO-NRs. In addition, the ZnO nanorods were shown to be slightly cytotoxic only at very high concentrations, while at 20 µg/mL no toxic effects were highlighted in those cells [33].

### 2.4. ROS Analysis

Reactive oxygen species (ROS) accumulation was evaluated in order to investigate whether ZnO-NRs-decorated GNPs may cause oxidative stress in oral pathogen bacteria. Indeed, several graphene-derived substrates with oxygen-containing functional groups may produce a surplus of ROS, which can contribute to the antimicrobial effect, but it could be also correlated to a higher cytotoxicity [44,45].

**Figure 8:**
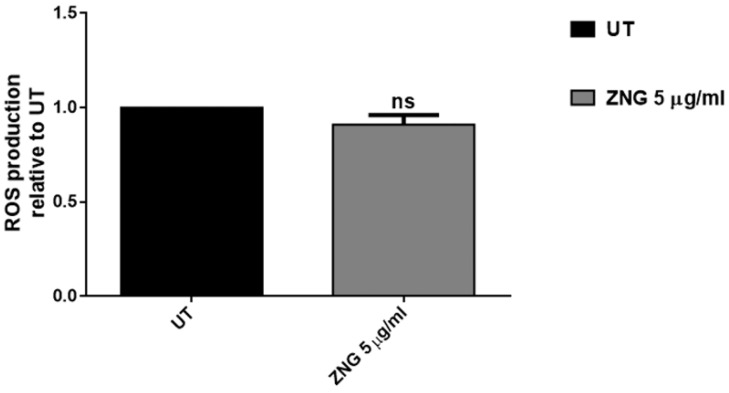
Cytoplasmic reactive oxygen species (ROS) content was evaluated by measuring the dichlorofuorescein diacetate (H_2_DCFDA) probe activation through ROS generation in *S. mutans* cells treated or not with 5 µg/mL suspensions of ZNGs for 2 h. Data are expressed as fluorescence relative to untreated cells. Statistical analysis was performed by Student’s *t*-test (ns not significant).

In the case of our nanomaterial, no ROS production was pointed out in treated cells, suggesting that oxidative stress did not participate to ZNG-induced cell death in *S. mutans* bacteria (Figure 8). It has been reported that non-oxidized nanoplatelets, including GNPs, did not generate ROS, even more highlighting their high biosafety and use as antimicrobial agents [20].

### 2.5. Inductively Coupled Plasma Mass Spectrometry Analysis

In order to assess if the antimicrobial properties could be due to the presence of zinc ions (Zn^2+^) released in the suspension by GNP-ZnO nanomaterial, we took advantage of the inductively coupled plasma mass spectrometry (ICP-MS) technique. The measurement of the Zn^2+^ release was thus performed in the supernatants derived from centrifugation of the ZNGs suspensions. The Zn^2+^ released by ZNGs was very low (i.e., 3.45 μg/mL) for the concentration corresponding to the maximum bactericidal action (50 μg/mL), and even became undetectable for the lowest one (0.1 μg/mL). Furthermore, we observed that the amount of zinc ions released by ZNGs was lower than pristine ZnO-NRs (i.e., 1.24 μg/mL vs. 2.58 μg/mL, respectively), when comparing suspensions having the same concentration of nanomaterial (5 μg/mL) (Table 1). Notably, upon incubation for 24 h with *S. mutans* cells, the suspensions of both ZNGs and ZnO-NRs showed lower levels of Zn^2+^ in comparison with cell-free suspensions (Table 1). Previous studies suggested that toxicity mechanisms of metal oxide-derived materials might be related to zinc ions release from nanoparticles as well as production of ROS, that can indirectly damage cell membranes through lipid peroxidation [46,47]. However, ROS generation was not detected in the case of ZNGs, as well as high amounts of released zinc ions, measured by ICP-MS technique.

Moreover, the lower zinc content measured into solutions after incubation with bacteria can be due to Zn “retention” by both/either cells and particles present into suspensions. However, the concentration of zinc ions obtained in all tested suspensions were shown to be remarkably lower in comparison with the minimum inhibitory concentration (MIC) reported for free Zn^2+^ against *S. mutans* cells [48,49]. Hence, it can be hypothesized that free Zn^2+^ do not contribute to the antimicrobial activity, which probably results from the mechanical interaction between ZNGs and the bacterial surface, as highlighted by the cell wall damages observed in FE-SEM analysis (Figure 7).

### 2.6. Bacterial Growth Inhibition by ZnO-NRs-decorated GNPs

Compared to other streptococci, *S. mutans* is considered as a highly cariogenic pathogen. This is mainly due to the fact that carbohydrates can ferment to lactic acid, formate, ethanol, and acetate [50]. *S. mutans* is generally acquired in oral cavity at the time of tooth eruption. However, as *S. mutans* has been detected in oral cavity of predentate children, the eruption of teeth seems not to be a necessary prerequisite, suggesting that this species may not be confined to dental plaque. In fact, it has been reported that *S. mutans* in planktonic lifestyle is able to adhere, invade, and survive within human gingival fibroblast cells [51].

**Figure 9:**
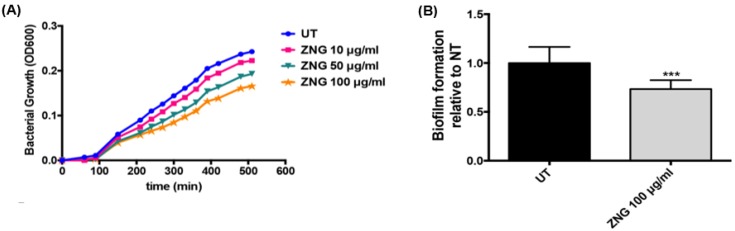
(**A**) Bacterial growth of *S. mutans* cells in media containing different concentrations of ZNGs is expressed as absorbance and OD600 was measured at the indicated time points; (**B**) Biofilm matrix was quantified by safranin binding assay. The production of EPS and biomass of *S. mutans* cells were evaluated after treatment with ZNGs and normalized to the untreated cells set as one. Statistical analysis was performed by Student’s t-test (*** *p* < 0.001 compared to the control).

In order to evaluate the effect on the bacterial growth, *S. mutans* cells were allowed to grow in media containing different concentrations of ZNGs under conditions inducing also biofilm formation, required for development of dental caries. Overall, GNP-ZnO nanocomposite affected the bacterial growth, as reported in Figure 9A, even if its effect is more marked at high concentrations; a 30% reduction was observed after 8 h. Indeed, biofilm formation of *S. mutans* in the presence of ZnO-NRs-decorated GNPs was also analyzed by evaluating biomass and exopolysaccharide (EPS) production, which are prerequisites for the formation and maintenance of bacterial biofilms. To this aim, the Safranin assay was performed and an almost 30% reduction of biofilm production was highlighted when challenging *S. mutans* with 100 µg/mL concentration of ZNGs with respect to the control (Figure 9B). This is in line with the notion that microorganisms in biofilms are more resistant to antibacterial agents than the planktonic form and much more concentrated biocide may be required for effective treatment [51]. In this case, the GNPs allow a more extended area that increases the nano-needles distribution, resulting in more efficient damage of a complex structure such as the biofilm.

Single and few-layer graphene coatings on SiO_2_ substrates were demonstrated to inhibit the bacterial adhesion [52]. Recently, it has been reported that *E. coli* cells treated with ZnO nanoparticles showed a strikingly high mortality rate of the cells due to a remarkable reduction in bacterial EPS, demonstrating that EPS can protect bacteria through sequestering nanoparticles [53]. Indeed, the reduction of safranin binding can be ascribed to a decreased amount of EPS, required for the biofilm formation and, in turn, for caries development. This may partly account for the killing effect exerted by ZNGs. We can speculate that EPS reduction may lead to lack of ZNG sequestering, thereby enhancing the nanostructure-specific bactericidal activity.

## 3. Materials and Methods

### 3.1. Materials

All chemicals were of reagent grade and used as received without further purification: graphite intercalation compound (GIC), zinc acetate dihydrate (Zn(CH_3_COO)_2_·2H_2_O, Sigma-Aldrich, ACS reagent, ≥98%, St. Louis, MO, USA), hexamine (C_6_H_12_N_4_, Fisher Scientific, ≥99%, Leicestershire, UK), and zinc nitrate hexahydrate (Zn(NO_3_)_2_·6H_2_O, Acros Organics, 98%, Geel, Belgium). The water used in all experiments was deionized (DI) and autoclave sterilized.

### 3.2. Production of Nanostructures and Suspensions

GNPs were produced by thermal expansion at 1050 °C for 30 s of commercially available Graphite Intercalation Compound (GIC), and successive liquid-phase exfoliation by probe sonication as described in Rago et al. (2015) [21]. Pristine ZnO-NRs were produced through the thermal decomposition of zinc acetate di-hydrate and successive probe sonication as described in our earlier works [32,34]. ZNGs were produced by directly growing ZnO-NRs onto unsupported GNPs in aqueous suspensions through a facile hydrothermal method [35].

Aqueous colloidal suspensions of either GNPs or ZnO-NRs or a mixing of GNPs and ZnO-NRs or ZNGs were prepared to evaluate the antibacterial activity through the dispersion of a defined amount of the powder like nanostructures in ultrapure and sterilized deionized water using probe sonication. The homogenous suspensions were then readily transferred to 50 mL sterilized centrifuge tubes.

Four different types of colloidal suspensions were prepared, with weight concentrations of the nanostructures ranging from 0.1 µg/mL up to 100 µg/mL, namely:

- suspensions of GNPs;
- suspensions of pristine ZnO-NRs;
- suspensions of mixture of both pristine GNPs and pristine ZnO-NRs;
- suspensions of ZnO-NRs-decorated GNPs (ZNGs).

### 3.3. Characterization of ZNGs

Samples for FE-SEM and EDX were prepared by drop-casting the suspensions containing the nanostructures onto cleaned silicon substrates, with subsequent drying in oven at 120 °C for 30 min.

Morphology and cell interaction investigations were carried out using a Zeiss Auriga FE-SEM available at SNN-Lab, operated at different accelerating voltages (varying between 2 and 5 keV) depending on the sample type.

The chemical elemental composition was investigated by EDX analysis equipped together with FE-SEM (Auriga, Zeiss, Oberkochen, Germany), and operated at 17 keV.

The crystalline structure and phase purity analysis was performed by X-ray diffraction using a Bruker (AXS D8-Advance) X-ray powder diffractometer equipped with incident-beam focusing X-ray mirrors and a position sensitive detector (Bruker AXS GmbH, Karlsruhe, Germany). Data were measured at room temperature, in transmission mode, using Cu Kα radiation (λ = 1.5418 Å, 40 kV at 40 mA), in a 2θ angular range ranging from 20° to 140° with a step size of 0.022° and 1 s of counting time. Samples were prepared as capillary mounts. Data were evaluated by the Rietveld method using Topas software [54].

### 3.4. Strains and Growth Culture

The strain utilized in this work was *Streptococcus mutans* ATCC 25175 and was grown in brain heart infusion broth (BHI) (Becton, Dickinson and Company, Franklin Lakes, NJ, USA) at 37 °C.

### 3.5. Cells Viability Test

About 5 × 10^7^ cells/mL were incubated in PBS (137 mM NaCl, 2.7 mM KCl, 10 mM Na_2_HPO_4_, 2 mM KH_2_PO_4_) at 37 °C with nanomaterials at the indicated concentrations under shaking for 24 h. Aliquots of samples were withdrawn, diluted, and then spread onto BHI agar plates (Becton, Dickinson and Company, Franklin Lakes, NJ, USA). After incubation at 37 °C, the capacity of the bacteria to form colonies was measured by counting the number of Colony Forming Units (CFU). Controls were run without nanomaterials suspensions.

### 3.6. FE-SEM Microscopy Imaging of Bacterial Cells

Scanning Electron Microscope investigation was carried out as aforementioned. Biological samples were prepared according to the procedures described earlier [20].

### 3.7. ROS Estimation

Dichlorofuorescein diacetate (H_2_DCFDA) dye (Thermo Fisher Scientific, Waltham, MA, USA) was used to assess the production of free intracellular radicals. Briefly, 5 × 10^7^ microbial cells treated or not for 2 h with ZnO-NRs or ZNGs suspensions, were washed with PBS and then incubated with 25 μM H_2_DCFDA for 30 min. The bacterial cells were washed with PBS twice and suspended in 500 µL di PBS. Afterwards, 200 µL of such suspensions were used to inoculate a 96-well microtiter plate. The amount of dichlorofluorescein (DCF) was measured as RFU by the microplate reader at 528 nm using an excitation at 485 nm.

### 3.8. Zn^2+^ Release

Zn-ion release in suspensions with different concentrations of ZNGs or with 5 µg/mL ZnO-NRs was measured by ICP-MS using a Perkin-Elmer SCIEX-ELAN 6100 equipped with a cross-flow nebulizer (Perkin-Elmer, Waltham, MA, USA). To this purpose, after sonication colloidal suspensions were allowed to settle for 24 h or incubated with *S. mutans* for 24 h at 37 °C. Then, the nanomaterials and/or cells were removed by two centrifugation steps (for 30 min at 1740× *g*). A blank procedure was always evaluated. Supernatants were analyzed after proper dilutions in 1% HNO_3_. A Zn ICP standard solution (MERCK, Darmstadt, Germany) of 1000 mg·L^−1^ in nitric acid was employed to prepare the calibrating solutions used to obtain the calibration curves, and a Rh ICP–MS standard solution (Aristar, BDH, Radnor, PA, USA) was used as the internal standard to correct matrix interferences in ICP-MS analysis.

### 3.9. Bacterial Growth Analysis

Bacterial growth (planktonic and biofilm bacteria) was evaluated by inoculating 10 µL of an overnight growth culture of *S. mutans* in a 96-well microtiter plate prepared as follows: each well was filled with 200 µL of Tryptic Soy Broth (TSB) (Becton, Dickinson and Company, Franklin Lakes, NJ, USA) with 5% sucrose containing, or not containing, different concentrations of ZNGs (in triplicate). Next, plates were incubated at 37 °C without agitation and the absorbance at 600 nm of each well was measured every 30 min.

### 3.10. Estimation of Biofilm Production

The safranin assay was used to evaluate biofilm mass and EPS production. Briefly, a suspension of overnight growth culture of *S. mutans* was diluted to 5 × 10^6^ cells/mL into fresh BHI with 5% sucrose containing, or not containing, different concentrations of ZNGs. Next, 200 µL of those solutions were used to inoculate 96-well microtiter plates. After incubation for 4 h at 37 °C, the medium was removed and biofilms were washed with PBS. Wells were stained with 0.1% safranin (Sigma-Aldrich, St. Louis, MO, USA) for 15 min, washed with PBS, and air-dried. Afterwards, 100 µL of 70% ethanol was added to dissolve biofilm and absorbance at 492 nm was then measured.

### 3.11. Statistical Analysis

Data are presented as mean ± SD, and Student’s *t*-test or one-way ANOVA analysis coupled with a Bonferroni post test (GraphPad Prism 5.0 software, GraphPad Software Inc., La Jolla, CA, USA) was used to determine the statistical significance between experimental groups. Statistical significance was defined as * *p* < 0.05, ** *p* < 0.01, and *** *p* < 0.001.

## 4. Conclusions

Our results indicated that ZNGs represent a powerful tool to kill both the planktonic and biofilm forms of *S. mutans*. The results reported in this paper lead us to consider that ZnO-NRs-decorated GNPs may be highly effective for controlling *S. mutans* growth and therefore caries development. 

Our data open new avenues for the improvement of resin composites and the associated dental adhesives utilizing graphene-derived material with promising antimicrobial properties.

## Figures and Tables

**Table 1:** Zn^2+^ concentration measured by ICP-MS in ZNG and ZnO-NRs suspensions incubated or not with *S. mutans* for 24 h.

Nanostructure Type	Nanostructure Concentration (µg/mL)	Treated with *S. mutans* Cells	Zn^2+^ Concentration (μg/mL)
ZnO-NRs	5	no	2.58
ZNGs	5	no	1.24
ZnO-NRs	5	yes	1.94
ZNGs	5	yes	0.61
